# Correction: Staining Pattern Classification of Antinuclear Autoantibodies Based on Block Segmentation in Indirect Immunofluorescence Images

**DOI:** 10.1371/journal.pone.0236463

**Published:** 2020-07-29

**Authors:** Jiaqian Li, Kuo-Kun Tseng, Zu Yi Hsieh, Ching Wen Yang, Huang-Nan Huang

## Notice of republication

Following publication of this article [1], concerns were raised about the availability of the underlying dataset and about the methodology used in this study.

The authors clarified that the original image database used in this study is available at 10.6084/m9.figshare.9928529. In the original database (consisting of 260 indirect immunofluorescence images categorized into four patterns), there were similarities and overlapping regions between some images assigned to the training set and the test set, which raised concerns about the suitability of the methodology and the reliability of the conclusions. In order to address these concerns, the authors carried out a supplemental reanalysis of a revised image database with similar and overlapping images removed. This notice reports the results of the supplemental reanalysis.

The authors identified and removed similar and overlapping images from the original database using locality preserving matching [2], the code for which is available at https://github.com/jiayi-ma/LPM. The revised database contains 166 images in total (92 coarse speckled (CS), 18 fine speckled (FS), 27 nucleolar (NU), and 29 peripheral (PE)). Full details of the methodology for removing similar and overlapping images are provided in S1 File of this notice. Details of the image files included in the original database, the image files removed and retained in the revised database, and their division into the revised training and test sets are provided in S2 File.

The image files included in the revised database employed for the supplemental reanalysis are available at 10.6084/m9.figshare.9928547.

A supplemental reanalysis of the revised database was carried out using the same algorithms from the original study. The full results of these supplemental reanalyses are provided in S3 File. Compared with the original results, the classification accuracy based on whole image decreases from 83.08% to 69.88%, and that based on cell segmentation also decreases from 90.77% to 84.34%. Moreover, the classification accuracy of these block images also reduces slightly, from 79.95% to 74.63% using local binary pattern (LBP) and Back Propagation Neural Network (BPNN), and from 82.21% to 78.72% using LBP and K-nearest neighbor (KNN). Nevertheless, the classification results of the whole image based on block segmentation using the method of LBP and KNN still remains stable. To verify this, a new table comparing the results of the classifications using the original image database and the revised image database is provided here as Table 7. The classification result from the reanalysis of the revised database using LBP, KNN, and weighted majority rule (WMR) increases slightly in contrast to the original database. The results using the combination rules of MR and WSR show a slight decrease, but performance remains above 90%. Overall, the classification performance using the method LBP and KNN is the best not only in the original database, but also in the revised database. This supports the article’s original conclusions.

**Table 7:** Comparison of classification results with two databases.

Method	Revised Database (166 samples)	Original Database (260 samples)
LBP+KNN+MR	90.36%	93.08%
LBP+KNN+WMR	95.18%	93.08%
LBP+KNN+WSR	92.77%	94.62%
LBP+BPNN+MR	87.95%	94.62%
LBP+BPNN+WMR	86.75%	93.87%

Differently, the classification results based on LBP and BPNN show a decrease as shown in Table 7, but they are still higher than those in the whole image classification and cell classification, consistent with the original conclusion. These decreases may be due to differences in their individual results for block image classification as mentioned above. The classification accuracy of these block images reduces from 79.95% in the original database to 74.63% in the revised database using LBP and BPNN.

A reanalysis of the original Fig 9, provided in S3 File, showed that compared with other methods, the combination of LBP and KNN achieves the best classification performance, in line with the conclusion based on the analysis of the original database. In addition, with the reduction of database size, the classification results based on other methods, such as grey-level co-occurrence matrix (GLCM), linear discrimination analysis (LDA) and scale-invariant feature transform (SIFT), decrease. This supports the robustness of this method in the classification of antinuclear antibody patterns.

To better validate the robustness and effectiveness of the methods, the training set and testing set were also divided randomly in the original database and revised database. Then LBP features were extracted from the database for KNN classifier. Please refer to the code file, ANA_Classification_LBP_KNN_Random_Rule.m (S4 File). 10 random classification results in the original and revised database are shown in Table 8. The classification performance remains stable in the original and revised databases, even though there are similar and overlapped images in the original database (260 samples). These experimental results support the stability and robustness of the methods.

**Table 8:** Classification results using random training and testing sets.

	1 (166 samples)			2 (260 samples)		
Method	LBP+KNN+MR	LBP+KNN+WMR	LBP+KNN+WSR	LBP+KNN+MR	LBP+KNN+WMR	LBP+KNN+WSR
1	93.98%	93.98%	93.98%	91.67%	93.18%	91.67%
2	91.57%	91.57%	92.77%	95.46%	95.46%	96.21%
3	91.57%	92.77%	96.38%	93.18%	93.94%	93.94%
4	91.57%	92.77%	92.77%	93.94%	93.94%	93.94%
5	91.57%	93.98%	95.18%	94.69%	96.21%	94.70%
6	90.36%	95.18%	93.98%	93.18%	95.45%	93.18%
7	93.97%	95.18%	91.57%	91.67%	95.45%	93.18%
8	92.77%	93.98%	95.18%	93.94%	93.94%	93.94%
9	95.18%	95.18%	96.39%	90.15%	91.67%	91.67%
10	91.57%	92.77%	93.98%	92.42%	93.18%	93.94%
Average	92.41%	93.74%	94.22%	93.03%	94.24%	93.64%

Finally, an additional 10x10 fold cross-validation both on the original data set and on the new reduced data set were performed. First, the dataset was averagely split into 10 subsets, with 9/10 used for training and the remaining 1/10 for testing, repeated for all ten subsets. Then, the above process was repeated 10 times for different subset assignments. The detailed results of classification based on LBP and KNN, LBP, and BPNN are shown in S5 File. To better compare the results, the average accuracy of 10x10 fold cross-validation was also calculated and is shown here in Table 9. The classification based on LBP and KNN was the best both on the original and revised datasets, and the classification results become better after removing the overlapped images from the original database. All these results in Table 9 are similar to the original results, further validating the efficiency and stability of the proposed methods. The codes for cross-validation are also included in S4 File. Please refer to ANA_Classification_LBP_BPNN_Random_Rule_100.m and ANA_Classification_LBP_KNN_Random_Rule_100.m.

**Table 9:** Comparison of average classification results with two databases in 10x10 cross-validation.

Method	Revised Database (166 samples)	Original Database (260 samples)
LBP+KNN+MR	95.08%	93.46%
LBP+KNN+WMR	95.12%	93.77%
LBP+KNN+WSR	95.80%	94.38%
LBP+BPNN+MR	89.81%	93.27%
LBP+BPNN+WMR	90.29%	93.15%

Some of the code published as Supporting Information on the original article [1] was re-used from other sources and is not offered under a CC-BY license and/or was not given clear attribution. The following codes were included:

- PCA, which is available at http://www.cad.zju.edu.cn/home/dengcai/Data/DimensionReduction.html
- GLCM_Feature1.m, which is available at https://www.mathworks.com/matlabcentral/fileexchange/22187-glcm-texture-features
- Vlfeat, which is available at http://www.vlfeat.org/
- HOG [3], which is available at https://www.mathworks.com/matlabcentral/fileexchange/28689-hog-descriptor-for-matlab
- getAllFiles.m, which is available at https://stackoverflow.com/revisions/2654459/3
- create_pr_net.m, which is available at https://github.com/ankitkala/Pattern-Recognition/blob/master/impact/create_pr_net.m

In light of concerns that the original license requirements were not met and/or permission to publish was not obtained for these codes, the original article [1] has been republished on July 10, 2020, with a revised version of Supporting Information files, in which the re-used code is replaced by the relevant links.

The Data Availability Statement has also been updated in the republished article as follows:

The underlying dataset of 260 images used in this study is available from https://doi.org/10.6084/m9.figshare.9928529. The authors confirm there are no restrictions on the use of these images. All other relevant data are within the paper and the Supporting Information files.

Please download this article again to view the correct version. Please note that the republished article incorporates only the changes to the Supporting Information Files and the updated Data Availability Statement. The reanalysis to address the methodological concerns is reported separately in this notice. The originally published uncorrected article and the republished, corrected article are provided here for reference (S6 and S7 Files).

## Supporting information

S1 File Database revision using locality preserving matching. (DOCX)

S2 File Image names in the original database, the removed database and revised database, as well as the revised training and testing datasets. (XLSX)

S3 File Reanalysed Tables and Figures using the revised database. (DOCX)

S4 File The updated code for the reanalysis. (ZIP)

S5 File The results of 10x10 fold cross-validation. (XLSX)

S6 File Originally published, uncorrected article. (PDF)

S7 File Republished corrected article. (PDF)
