# Supplementary material for: Staining Pattern Classification of Antinuclear Autoantibodies Based on Block Segmentation in Indirect Immunofluorescence Images
Source: PLoS One. 2014 Dec 4;9(12):e113132. doi: 10.1371/journal.pone.0113132 (PMC4256175; doi:10.1371/journal.pone.0113132)
Supplement: Code S1 — (ZIP) [file pone.0113132.s002.zip › Code Instructions.docx]

**Code Instructions**

The experiment can be divided into three parts, cell-level classification, block-level classification and specimen-level (image-level) classification. The block-level classification is the main part, and the other two are regarded as the control groups.

# Block-Level Classification

The source codes of block-level classification are described as follows. We should firstly segment the original image into various blocks in the source file of ANA_Seperation_Extraction_multidivisions.m, then extract the different LBP operators in ANA_Block_Seperation_LBP_Extraction.m, and calculate the number of cells in the single blocks, and finally classify the staining pattern in the other source files with different classifiers, such as KNN in ANA_Classification_LBP_KNN_Combination_Rule.m. As for other features (GLCM, LDA, PCA, SIFT), the feature extraction and classification are combined in one file, such as GLCM feature and SVM classifier in ANA_Classification_GLCM_SVM_Combination_Rule.m. The remaining files are functions used in the other script files.

- LBP：Matlab code of Local Binary Pattern algorithm
- ANA_Seperation_Extraction_multidivisions.m: block segmentation
- ANA_Cell_Number_Calculation.m: calculate the number of cells in a block
- ANA_Block_Seperation_LBP_Extraction.m: extract the LBP feature
- ANA_Classification_GLCM_BPNN_Combination_Rule.m: classification with GLCM feature and BPNN classifer
- ANA_Classification_GLCM_KNN_Combination_Rule.m: classification with GLCM feature and KNN classifer
- ANA_Classification_GLCM_SVM_Combination_Rule.m: classification with GLCM feature and SVM classifer
- ANA_Classification_LBP_BPNN_Combination_Rule.m: classification with LBP feature and BPNN classifer
- ANA_Classification_LBP_KNN_Combination_Rule.m: classification with LBP feature and KNN classifer
- ANA_Classification_LBP_SVM_Combination_Rule.m: classification with LBP feature and SVM classifer
- ANA_Classification_PCA_BPNN_Combination_Rule.m: classification with PCA feature and BPNN classifer
- ANA_Classification_PCA_KNN_Combination_Rule.m: classification with PCA feature and KNN classifer
- ANA_Classification_PCA_LDA_Combination_Rule.m: classification with LDA feature and KNN classifer
- ANA_Classification_SIFT_vlfeat_Combination_Rule.m: classification with SIFT feature in VLFeat package
- decideKNNReliability.m: to calculate the reliability of KNN
- decideOverlap2.m: to decide whether two blocks overlap
- decideRemovedBlock.m: to decide the blocks should be removed
- decideZeroInTwo.m: to determine whether there are no overlaps after removing some blocks
- movingWindowLocation2.m: function to segment blocks from original images
- crossCorrelationMatrix.m: function to calculate the cross correlation

# Cell-Level Classification

- LBP：Matlab code of Local Binary Pattern algorithm
- ANA_Cells_Seperation_GLCM_Extraction.m：to extract the GLCM feature
- ANA_Cells_Seperation_GLCM_HOG_Extraction.m：to extract the GLCM and HOG feature
- ANA_Cells_Seperation_LBP_Extraction.m：to extract the LBP feature
- ANA_Cells_GLCM_HOG_SVM_Combination_Rule.m：classification based on cell segmentation with GLCM and HOG feature and SVM classifier
- ANA_Cells_GLCM_KNN_Combination_Rule.m: classification based on cell segmentation with GLCM feature and KNN classifier
- ANA_Cells_GLCM_SVM_Combination_Rule.m: classification based on cell segmentation with GLCM feature and SVM classifier
- ANA_Cells_LBP_KNN_Combination_Rule.m: classification based on cell segmentation with LBP feature and KNN classifier
- ANA_Cells_LBP_SVM_Combination_Rule.m: classification based on cell segmentation with LBP feature and SVM classifier
- decideKNNReliability.m: function to calculate the reliability of KNN
- separateANACells.m: cell segmentation

Similar to the block-level classification, firstly locate and segment the HEp-2 cells in the original images and then extract the different features of these cells, that is, GLCM feature in ANA_Cells_Seperation_GLCM_Extraction.m, GLCM and HOG feature in ANA_Cells_Seperation_GLCM_HOG_Extraction.m and LBP feature in ANA_Cells_Seperation_LBP_Extraction.m. And subsequently we classify the staining patterns of cells by different classifiers and the staining patterns of images by different fusion rules in these files, such as ANA_Cells_GLCM_HOG_SVM_Combination_Rule.m, ANA_Cells_LBP_SVM_Combination_Rule.m etc.

# Image-Level Classification

This part is significantly easier than other two parts, that is, we need not to segment the blocks or cells in these images in the image-level classification. We directly extract the features of the original images (HOG, GLCM, LBP and PCA) and then classify the staining patterns of these images using KNN classifier and SVM classifier.

- LBP：Matlab code of Local Binary Pattern algorithm
- ANA_Whole_Image_HOG_GLCM_KNN_Method.m: classification in image level with HOG and GLCM feature and KNN classifier
- ANA_Whole_Image_HOG_GLCM_SVM_Method.m: classification in image level with HOG and GLCM feature and SVM classifier
- ANA_Whole_Image_LBP_PCA_KNN_Method.m: classification in image level with LBP feature and KNN classifier
- ANA_Whole_Image_PCA_KNN_Method.m: classification in image level with PCA feature and KNN classifier

Codes used from other links:

- PCA: <http://www.cad.zju.edu.cn/home/dengcai/Data/DimensionReduction.html>
- GLCM_Feature1.m: <https://www.mathworks.com/matlabcentral/fileexchange/22187-glcm-texture-features>
- Vlfeat : <http://www.vlfeat.org/>
- Libsvm : <https://www.csie.ntu.edu.tw/~cjlin/libsvm/index.html>
- HOG: <https://www.mathworks.com/matlabcentral/fileexchange/28689-hog-descriptor-for-matlab>
- getAllFiles.m: <https://stackoverflow.com/revisions/2654459/3>
- create_pr_net.m: <https://github.com/ankitkala/Pattern-Recognition/blob/master/impact/create_pr_net.m>
