# Supplementary material for: Correction: Staining Pattern Classification of Antinuclear Autoantibodies Based on Block Segmentation in Indirect Immunofluorescence Images
Source: PLoS One. 2020 Jul 29;15(7):e0236463. doi: 10.1371/journal.pone.0236463 (PMC7390282; doi:10.1371/journal.pone.0236463)
Supplement: S1 File — (DOCX) [file pone.0236463.s001.docx]

**Database revision using locality preserving matching**

To efficiently remove the similar and overlapped images, we used locality preserving matching to detect the matching degree of two images. Locality preserving matching is an image matching approach that can remove mismatches from given putative image feature correspondences and simultaneously maintain the local neighborhood structures of those potential true matches. The method used a mathematical model, and derived a closed-form solution with linearithmic time and linear space complexities. First, a set of putative matches is constructed by considering all possible matches between two feature sets and filtering out matches whose feature descriptor vectors are sufficiently different. Then a geometric constraint is used to remove the false matches contained in the putative set, which further filters out those matches with different spatial neighborhood structures among feature points. The based putative correspondence between feature sets can be efficiently established using the well-designed feature SIFT descriptors. More importantly, the locality preserving matching has been demonstrated to be useful in the image matching in the medical image analysis, for example, Retina dataset. For detail methodology, please refer to the original article,

Ma, J., Zhao, J., Jiang, J., Zhou, H., & Guo, X. (2019). Locality preserving matching. International Journal of Computer Vision, 127(5), 512-531. <https://link.springer.com/article/10.1007/s11263-018-1117-z>

Also, the corresponding code is also provided online, <https://github.com/jiayi-ma/LPM>

To better remove the similar and overlapped images, we used the recall rate to measure the similarity degree of two images. Here the recall rate is defined as the ratio of the number of correct matches to the number of correspondences:

$$recall rate=\frac{the number of correct matches}{the number of correspondences}$$

To demonstrate whether the recall rate is suitable parameter for our analysis, we firstly compared their recall rates between two similar (Figure 1, P5030906.JPG and P5030907.JPG), overlapped (Figure 2, P5037395.JPG and P5037396.JPG), or different images (Figure 3, P5030906.JPG and P5030908.JPG). Their recall rates are 68.41%, 74.03% and 0.85%, respectively. Obviously, the recall rates between two similar or overlapped images are greatly larger than that between two different images. Thus, the recall rate can be utilized to determine the similarity degree between two images.


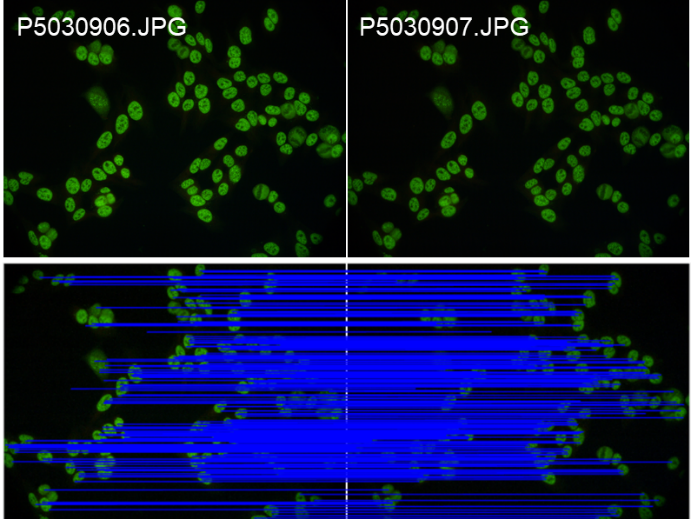


**Figure 1** Feature matching results of two similar images. The blue lines indicate the positions of the matching points in two images. For visibility, in the image pairs, at most 300 randomly selected matches are presented. The recall rate between two images is 68.41%.


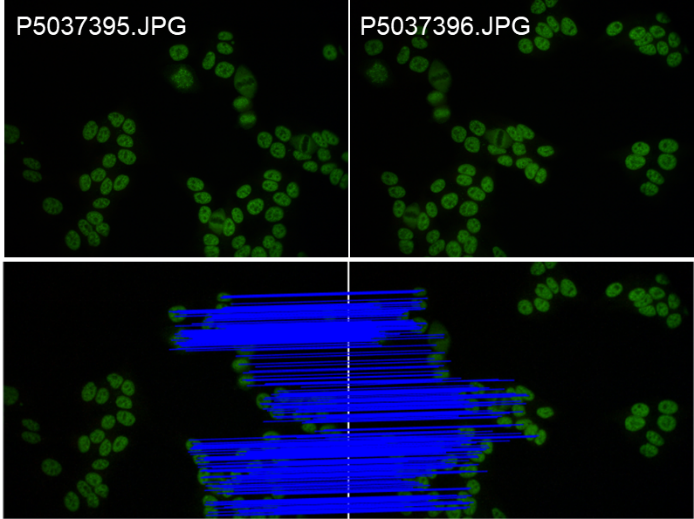


**Figure 2** Feature matching results of two overlapped images. The blue lines indicate the positions of the matching points in two images. For visibility, in the image pairs, at most 300 randomly selected matches are presented. The recall rate between two images is 74.03%.


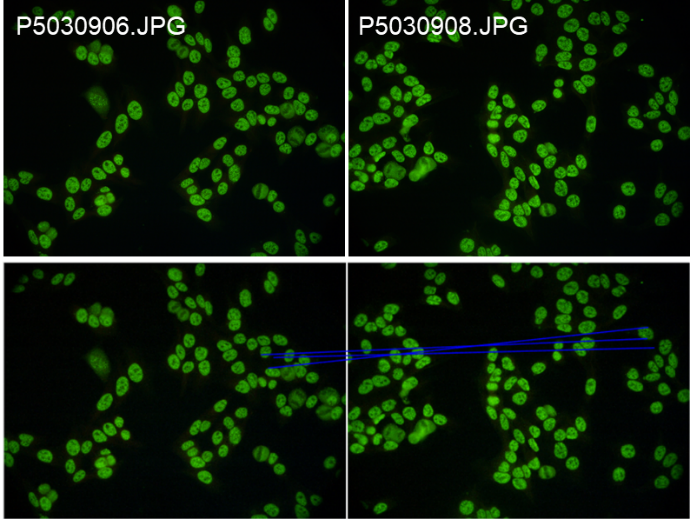


**Figure 3** Feature matching results of two different images. The blue lines indicate the positions of the matching points in two images. For visibility, in the image pairs, at most 300 randomly selected matches are presented. The recall rate between two images is 0.85%.

To remove more similar or overlapped images, but simultaneously remain enough images for our classification analysis, a proper threshold is needed to determine whether one of two similar or overlapped images need to be removed or not. Here we chose the average maximum recall rate of all images in the original database, and the threshold is set as *T* = 36.13%. When the recall rate of two images is above than *T*, one of two images would be removed. In this way, 94 similar or overlapped images are removed, and their average maximum recall rate is 69.63%. For detail removed images, please see the Supporting File S2. It should be noted that if the threshold *T* is set to be small, there will not be enough images in the new database for further analysis. Thus, here we aim to remove the most similar or overlapped images with relatively largest recall rates, and at the same time remaining more images for image classification. In addition, the image overlapping is a common phenomenon in the pattern classification and machine learning, for example, face recognition and fingerprint recognition. When the dataset has limited data, especially in the medical image analysis, data augmentation algorithms are needed to enrich the database, such as geometric transformations, color space augmentations, kernel filters, mixing images, random erasing and feature space augmentation. The new databases obtained by using these data augmentation algorithms inevitably has similarity or overlaps between different images. For more information, please see the reference,

Shorten, C., & Khoshgoftaar, T. M. (2019). A survey on Image Data Augmentation for Deep Learning. Journal of Big Data, 6(1), 60.

To check whether the remaining image dataset contains no similarities and overlaps or not, we calculated the average recall rate and average number of matching points in the revised dataset. The average recall rate of the remaining images is about 3.08% and the average number of matching points in the remaining dataset is 6.8557. That means there is only about 7 points that can correctly match between two images with a size of 3136x2352 pixel. This is an understandable and acceptable measurement because there are some matching points even between two random different images. So far, we demonstrated that the revised dataset has almost no large overlaps between two images.
