# Supplementary material for: Correction: Staining Pattern Classification of Antinuclear Autoantibodies Based on Block Segmentation in Indirect Immunofluorescence Images
Source: PLoS One. 2020 Jul 29;15(7):e0236463. doi: 10.1371/journal.pone.0236463 (PMC7390282; doi:10.1371/journal.pone.0236463)
Supplement: S3 File — (DOCX) [file pone.0236463.s003.docx]

**The Reanalysed Tables and Figures**

This document presents the results of the supplemental reanalyses using a revised image dataset.

Reanalysis of Table **1**: The samples in the training set and in the testing set

| IIF patterns | Samples in the training set | Samples in the testing set |
| --- | --- | --- |
| Coarse speckled | 46 | 46 |
| Fine speckled | 9 | 9 |
| Nucleolar | 14 | 13 |
| Peripheral | 14 | 15 |
| Total | 83 | 83 |

Reanalysis of Table **2**: Classification results based on the whole image

|  | CS | FS | NU | PE | Accuracy |
| --- | --- | --- | --- | --- | --- |
| CS | 37 | 0 | 4 | 5 | 80.43% |
| FS | 0 | 9 | 0 | 0 | 100.0% |
| NU | 4 | 0 | 9 | 0 | 69.23% |
| PE | 8 | 1 | 3 | 3 | 20.00% |
| Total |  |  |  |  | 69.88% |

Reanalysis of Table **3**: Classification results based on cell segmentation with KNN classifier, LBP feature and Majority Rule

|  | CS | FS | NU | PE | Accuracy |
| --- | --- | --- | --- | --- | --- |
| CS | 45 | 0 | 1 | 0 | 97.83% |
| FS | 0 | 7 | 2 | 0 | 77.78% |
| NU | 7 | 1 | 5 | 0 | 38.46% |
| PE | 2 | 0 | 0 | 13 | 86.67% |
| Total |  |  |  |  | 84.34% |

Reanalysis of Table **4**: Classification results of block pattern classification of LBP+KNN and LBP+BPNN

| Block patterns | Ntrain | Ntest | Nc (LBP+BPNN) | Nc (LBP+KNN) |
| --- | --- | --- | --- | --- |
| CS | 893 | 867 | 719(82.93%) | 752(86.73%) |
| FS | 123 | 107 | 88(82.24%) | 105(98.13%) |
| NU | 313 | 279 | 177(63.44%) | 212(75.99%) |
| PE | 324 | 312 | 184(58.97%) | 163(52.24%) |
| Total | 1653 | 1565 | 74.63% | 78.72% |

Ntrain: number of blocks in the training set

Ntest: number of blocks in the testing set

Nc**:** number of correct classification of blocks

Reanalysis of Table **5**: Classification results based on block segmentation with KNN classifier, LBP feature and Weighted Majority Rule

|  | CS | FS | NU | PE | Accuracy |
| --- | --- | --- | --- | --- | --- |
| CS | 45 | 0 | 1 | 0 | 97.83% |
| FS | 0 | 9 | 0 | 0 | 100.0% |
| NU | 0 | 0 | 13 | 0 | 100.0% |
| PE | 3 | 0 | 0 | 12 | 80.00% |
| Total |  |  |  |  | 95.18% |

Reanalysis of Table **6**: Comparison of direct whole image classification, classification based on cell segmentation and classification based on block segmentation

|  | Total accuracy | Mean class accuracy |
| --- | --- | --- |
| Direct whole image classification | 69.88% | 67.42% |
| Cordelli E[34] (LBP and KNN) and WSR | 90.77% | 78.14% |
| Cordelli E[34] (LBP and KNN) and MR | 90.00% | 79.03% |
| Cordelli E[34] (LBP and SVM) and MR | 63.85% | 25.00% |
| Ghosh S [24] (Texture feature, HOG and SVM) and MR | 58.46% | 22.89% |
| Di Cataldo S [16] (GLCM and SVM) and MR | 59.23% | 23.19% |
| Classification based on block segmentation | 95.18% | 94.46% |


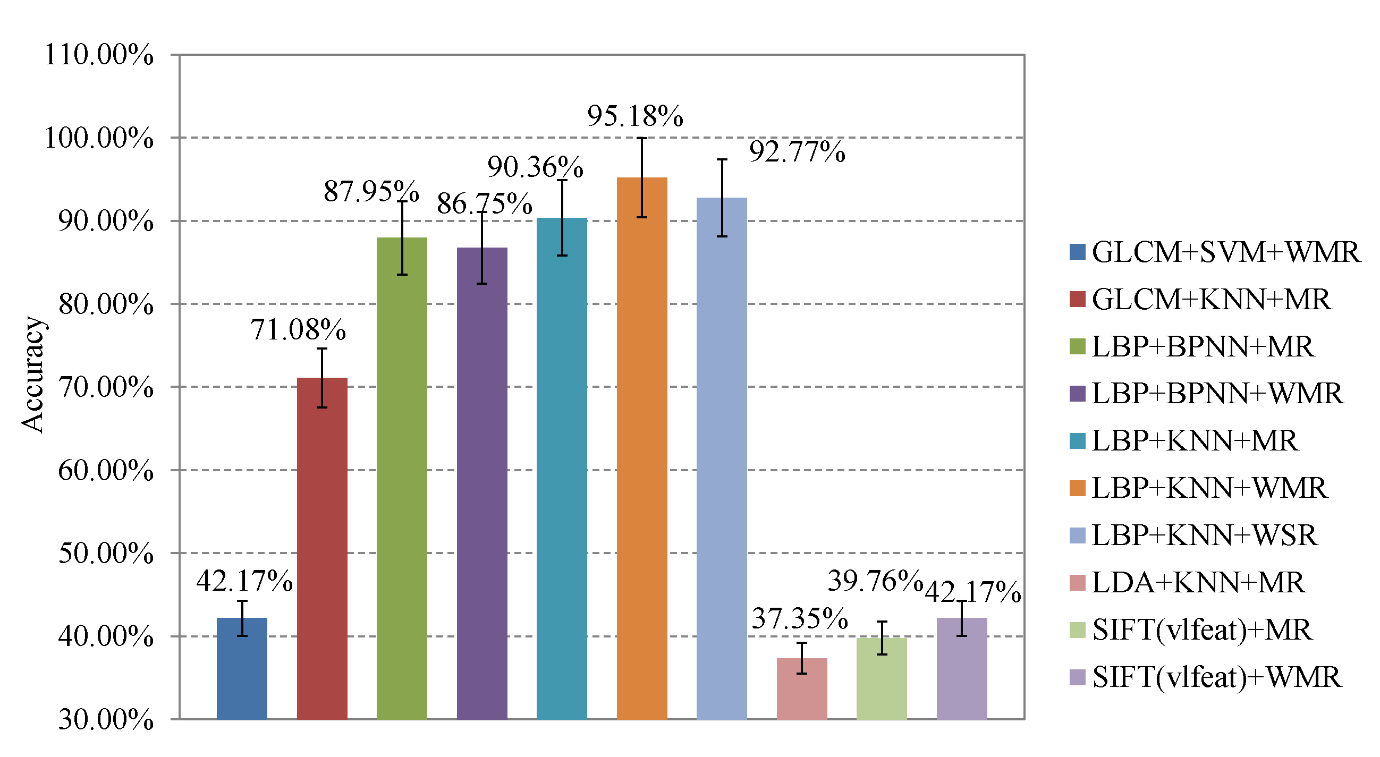


**Reanalysis of Figure 9**. Accuracies of different combinations of classifier, feature and fusion rule using the revised database: from right to left sequentially GLCM+SVM+WMR, GLCM+KNN+MR, LBP+BPNN+MR, LBP+BPNN+WMR, LBP+KNN+MR, LBP+KNN+WMR, LBP+KNN+WSR, LDA+KNN+MR, SIFT(vlfeat)+MR and SIFT(vlfeat)+WMR.
