# Supplementary material for: Correction: Staining Pattern Classification of Antinuclear Autoantibodies Based on Block Segmentation in Indirect Immunofluorescence Images
Source: PLoS One. 2020 Jul 29;15(7):e0236463. doi: 10.1371/journal.pone.0236463 (PMC7390282; doi:10.1371/journal.pone.0236463)
Supplement: S7 File — (PDF) [file pone.0236463.s007.PDF]

RESEARCH ARTICLE

Staining Pattern Classification of Antinuclear Autoantibodies Based on Block Segmentation in Indirect Immunofluorescence Images

Jiaqian Li[1], Kuo-Kun Tseng[1]*, Zu Yi Hsieh[2], Ching Wen Yang[3], Huang-Nan Huang[4]

1. Department of Computer Science and Technology, Harbin Institute of Technology, Shenzhen Graduate School, Shenzhen, China, **2.** Department of Internal Medicine, Taichung Veterans General Hospital, Taichung, Taiwan, **3.** Computer & Communication Center, Taichung Veterans General Hospital, Taichung, Taiwan, **4.** Department of Mathematics, Tunghai University, Taichung, Taiwan

*kktseng@foxmail.com

OPEN ACCESS

Citation: Li J, Tseng K-K, Hsieh ZY, Yang CW, Huang H-N (2014) Staining Pattern Classification of Antinuclear Autoantibodies Based on Block Segmentation in Indirect Immunofluorescence Images. PLoS ONE 9(12): e113132. doi:10.1371/journal.pone.0113132

Editor: Hans A. Kestler, University of Ulm, Germany

Received: April 28, 2014

Accepted: October 20, 2014

Published: December 4, 2014

Copyright: © 2014 Li et al. This is an open-access article distributed under the terms of the Creative Commons Attribution License, which permits unrestricted use, distribution, and reproduction in any medium, provided the original author and source are credited.

Data Availability: The underlying dataset of 260 images used in this study is available from https://doi.org/10.6084/m9.figshare.9928529. The authors confirm there are no restrictions on the use of these images. All other relevant data are within the paper and the Supporting Information files.

Funding: The authors have no support or funding to report.

Competing Interests: The authors have declared that no competing interests exist.

Abstract

Indirect immunofluorescence based on HEp-2 cell substrate is the most commonly used staining method for antinuclear autoantibodies associated with different types of autoimmune pathologies. The aim of this paper is to design an automatic system to identify the staining patterns based on block segmentation compared to the cell segmentation most used in previous research. Various feature descriptors and classifiers are tested and compared in the classification of the staining pattern of blocks and it is found that the technique of the combination of the local binary pattern and the k-nearest neighbor algorithm achieve the best performance. Relying on the results of block pattern classification, experiments on the whole images show that classifier fusion rules are able to identify the staining patterns of the whole well (specimen image) with a total accuracy of about 94.62%.

Introduction

Autoimmune diseases, such as rheumatoid arthritis, primary biliary cirrhosis and dermatomyositis, are individually rare in contrast with other kinds of diseases, but together they affect the health of many people worldwide. They are a fascinating but poorly understood group of diseases [1]. Antinuclear autoantibodies are a serological hallmark of most autoimmune diseases, and serve as diagnostic biomarkers and classification criteria for a number of these diseases [2]. Although the role of autoantibodies is still not clear, growing evidence shows that most

autoimmune diseases are confirmed to be in connection with the occurrence of specific auto-antibodies, such as primary biliary cirrhosis [3]. However, antinuclear antibodies are also detectable in approximately 50% of subjects with primary biliary cirrhosis. Several ANAs are associated with primary biliary cirrhosis, so the connection of a specific ANA to the pathogenesis of primary biliary cirrhosis is not known [3]. This demonstrates that the relationship between autoimmune diseases and autoantibodies is not a single correspondence.

Although there are many tests for the detection of ANAs, such as indirect immunofluorescence (IIF) and enzyme-linked immunosorbent assay (ELISA), IIF based on HEp-2 cell substrate during the serological hallmark is the most commonly used staining method for antinuclear autoantibodies. Usually, the immunofluorescence patterns are manually identified by the physician visually inspecting the slides under a microscope. Since IIF diagnosis requires both the estimation of fluorescence intensity and the description of staining patterns, adequately trained persons are not always available for these tasks, so this procedure still needs highly specialized and experienced physicians to make the diagnoses. As ANA testing becomes more used in clinics, an automatic inspection system for pattern categories is in great demand [4].

Before the classification of staining patterns, relevant patterns (see Figure 1) related to the most recurrent ANAs should be considered [5, 6] in the experimental dataset.

- *Coarse Speckled:* this pattern is characterized by coarse granular nuclear staining of the interphase cell nuclei;
- *Fine Speckled:* this pattern is characterized by fine granular nuclear staining of the interphase cell nuclei;
- *Peripheral:* this group is characterized by solid staining, primarily around the outer region of the nucleus, with weaker staining toward the centre of the nucleus;
- *Nucleolar:* this pattern is characterized by large coarse speckled staining within the nucleus, less than six in number per cell.

The aim of this paper is to design an automatic system with a two-layer classification model, block pattern recognition and well pattern recognition, to identify the staining patterns of the whole well based on block segmentation. In particular, the following points will be investigated in the present study:

1) *Block segmentation.* In contrast to the previous cell segmentation used for ANA classification, block segmentation is significantly easier to implement and more applicable due to the erroneous conditions of cell segmentation.
2) *Block pattern classification.* Various image features (local binary pattern (LBP), linear discrimination analysis (LDA), scale-invariant feature transform (SIFT) and grey-level co-occurrence matrix (GLCM) and classifiers K-nearest neighbour (KNN), Back Propagation Neural Network (BPNN) and support vector machine (SVM) are compared in this step to seek the best characteristic and classifier for ANA classification.

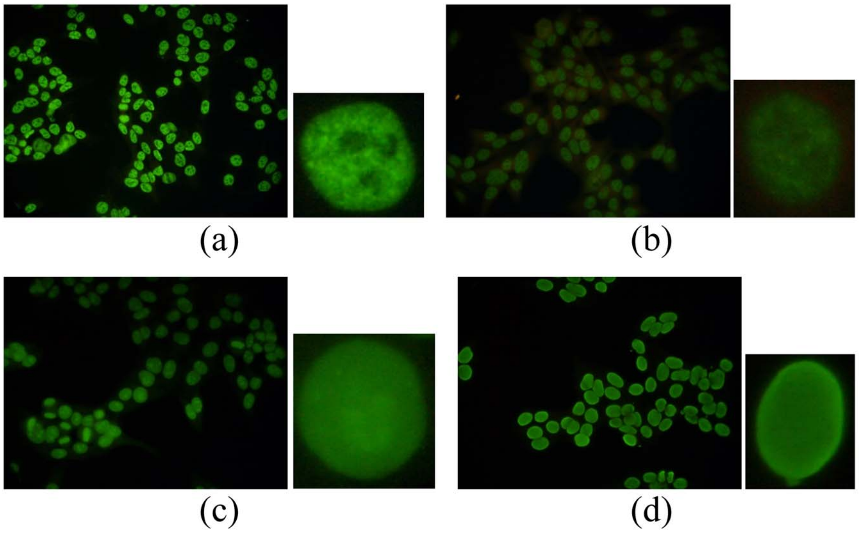

Figure 1. ANA patterns in the experimental dataset: (a) coarse speckled (b) fine speckled (c) nucleolar (d) peripheral.

doi:10.1371/journal.pone.0113132.g001

3) *Well pattern classification.* Based on the results of the block pattern classification, classifier fusion rules are used to identify the staining patterns of the whole well. Meanwhile, a kind of cell pattern classification is regarded as the control group.

The rest of this paper comprises four parts. In Section 2, we introduce some related studies on ANA patterns including segmentation, feature extraction and classification. Section 3 presents the proposed method consisting of four steps: block segmentation, feature extraction, block pattern classification and well pattern classification. Section 4 provides the experimental results and comparison. Finally Section 5 is the conclusion and discussion.

Related Studies

2.1 Image Segmentation

The previous research on ANA image segmentation has mainly focused on cell segmentation and the criteria for recognition of cell patterns, but a more applicable method of block segmentation for ANA pattern classification has so far not been developed. Many competitions and conferences research cell classification and cell segmentation, for example, the competition on cell classification by fluorescent image analysis hosted by the 20th IEEE International Conference on Image Processing (ICIP) and The 1st Workshop on Pattern Recognition Techniques for Indirect Immunofluorescence Images.

Creemers et al. [7] repeatedly used image processing techniques, including morphological opening and Otsu thresholding, to cut out the needed region of

interest. It was found that this method has the capability to split connected regions into individual cells with an average accuracy of 89.57%.

Huang et al. [8] proposed an efficient method for automatically detecting the outlines of fluorescent cells in IIF images. This method first classified an IIF image into two cases, sparse and mass cell regions, based on the number of connected regions in an image. Depending on the cell types of the images, different colour spaces and processing techniques were adopted for cell segmentation. For images with sparse region cells, HSB colour space, anisotropic diffusion, canny edge detection and morphological smoothing are applied sequentially to detect the cell outline, while for images with mass region cells, CMY colour space, anisotropic diffusion, Otsu's thresholding and morphological processing are used.

Hsieh et al. and Huang et al. [9, 10] also presented a reliable region-based method of two-staged watershed segmentation to solve a wide range of difficult problems of ANA image segmentation, i.e. over-segmentation and sensitivity to noise and contrast in the image. Region merging and region elimination were utilized for the first stage watershed algorithm [11] to obtain the cell boundaries and in the second stage the similarity-based watershed algorithm acted as the marker to prevent over-segmentation. It was proved that the segmentation performance achieved an overall sensitivity of 94.7%.

2.2 Image Feature Extraction

Numerous features utilized in ANA pattern classification were investigated, including texture features and shape features, as shown in Figure 2. Since the same object may have a variety of different colours but a similar shape, many queries may arise as to the shape of the image instead of the colour of the image. There are two methods of presenting shape features: contour feature and regional characteristics. However, shape features lack a model, and have high computation and storage requirements. In [12], the shape measurement of a single feature vector, with greater weight by far given to texture, is used to identify the cytoplasmatic class and the shape feature (calculated as the area divided by the square of the perimeter) is able to recognize most samples of this category based on a single parameter. In [4], four shape features, area, perimeter, inside area and perimeter area, in the feature vector are utilized as the inputs for a self-organizing map (SOM) model to determine the similarity of the cells.

Texture feature is the most commonly used feature for pattern classification, describing the surface nature of the scene corresponding to a specified image or image area. Texture feature is not the sort of feature based on pixels, which need statistical calculations of more than one pixel belonging to the region. As a statistical feature, texture feature, often with rotation-invariant characteristics, has a strong capability to resist noise. However, it also has its drawbacks, and one obvious drawback is that changing the image resolution, may result in larger deviations in the calculated texture feature. Moreover, the feature may sometimes be affected by light and reflection. Texture feature extraction methods can be divided into statistical methods, structure methods and spectrum methods.

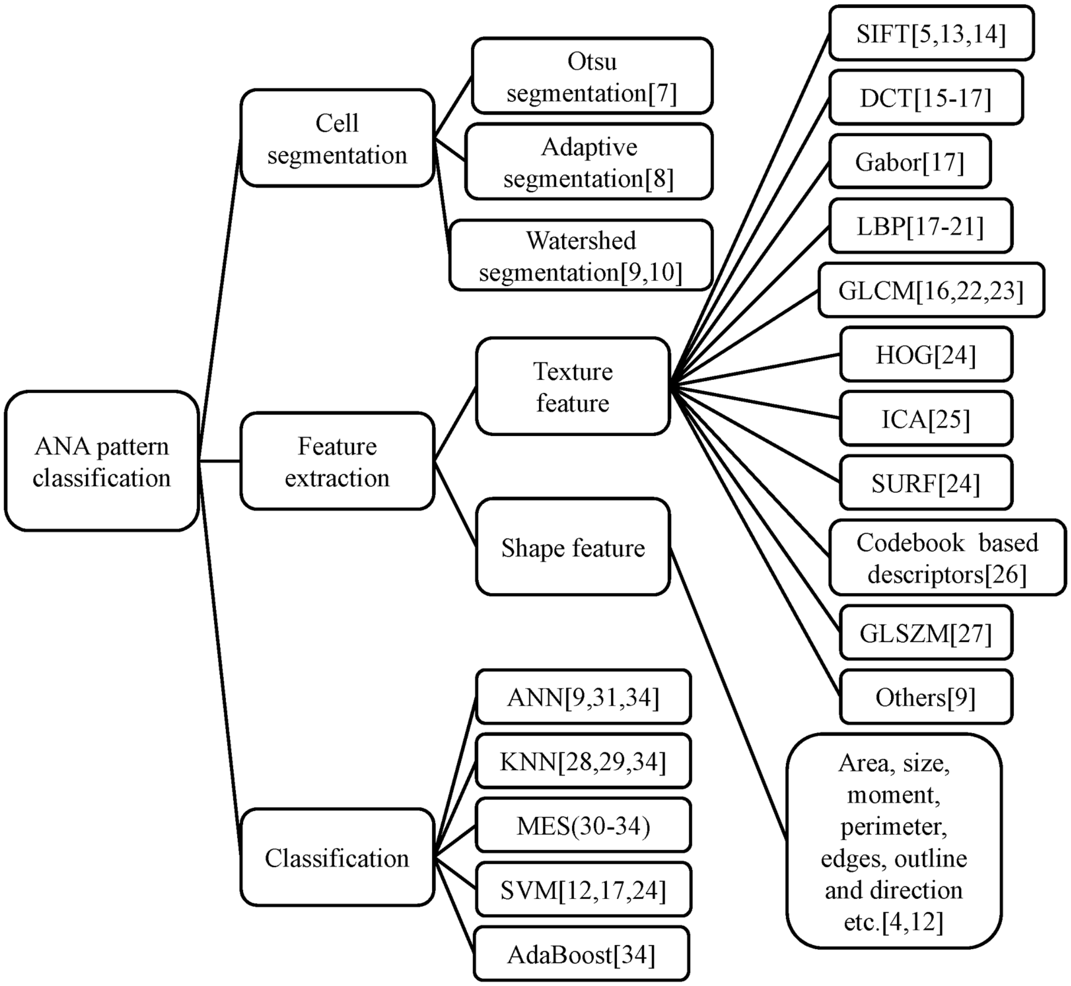

Figure 2. ANA pattern classification methods: segmentation, feature extraction and classification.

doi:10.1371/journal.pone.0113132.g002

Giulio et al. [5] used the well-known SIFT [13, 14] descriptor to extract concise and informative local characteristic from HEp-2 images. The SIFT algorithm has proved to be one of the most effective in the object-recognition field because of its invariance to common image transformations, illumination changes and noise. Discrete cosine transform (DCT) [15] is always used to extract relevant textural information for image compression and classification. In [16], 328 DCT coefficients, which represent different patterns of image variation and directional information of the texture, are calculated through two-dimensional DCT of the normalized images. In [17], 48 DCT features, including the DC component, mean value and standard variance, are extracted for HEp-2 cell pattern classification. The LBP descriptor [18–20] is a robust and computationally efficient means of texture description, which derives from a general definition of texture in a local neighbourhood, incorporates both statistical and structural information and has shown effectiveness in many applications. Kuan et al. [17] extracted 42 features

from each HEp-2 cell image through the multi-resolution LBP descriptors. Ersoy et al. [21] used a uniform rotation-invariant LBP consisting of 18 unique patterns for HEp-2 cell classification.

GLCM [22] is a powerful technique that extracts texture characteristics from the spatial relationship among intensity values at specified offsets and reports the distribution of co-occurring values among local pixels based on different distances and angles. In [23], only four GLCM features (intensity, standard deviation, entropy and range) are calculated as a part of the final feature vector, while in [16], a total number of 44 features, represented by the mean and the range value over the 22 statistical measures (e.g. correlation, cluster prominence, cluster shade, energy, entropy, variance, homogeneity, maximum probability, etc. [22]), are extracted from four GLCMs.

There are many other texture features used for HEp-2 cell classification, such as Gabor transform [17], histogram of oriented gradients (HOG) [24], independent component analysis (ICA) [25], codebook based descriptors [26], speeded-up robust features (SURF) [24], grey-level size zone matrix (GLSZM) [27] etc. However, most studies combine several of the image features mentioned above into a feature vector to recognize the patterns of the HEp-2 cell instead of using a single characteristic since a combination of several features is able to extract more image information on texture, shape and space than a single feature. In [24], Ghosh and Chaudhary. explored various features like SURF in a bag of words (BoW) model, texture-based features from the GLCMs and region of interest (ROI)-based features and HOG features, using one or several of them to create a composite feature vector to investigate the performance of a classification based on various features. A total of 372 features containing 44 GLCM features and 328 DCT coefficients in [16] were used to characterize each HEp-2 image. Moreover, in [9] a total of eight practical features (standard deviation, uniformity/entropy, block variation of local correlation coefficients, spatial grey-level dependence matrices, grey-level difference matrix, neighbourhood grey-tone difference matrix, fractal dimension and image coarse degrees) obtained from an IIF cell image were utilized to identify fluorescence patterns.

2.3 ANA Pattern Classification

In the past decade, there have been many studies on the detection of ANA patterns and many classification methods, mainly including the KNN algorithm [28, 29], artificial neural networks (ANNs), expert systems (ESs) and SVM etc., have been utilized for pattern recognition in HEp-2 cells and have achieved positive final performance. Multi-class SVMs with different kernels were investigated and used in [12, 17, 24]. Soda has always used multi-expert systems (MESs) [30–33] to explore the problem of HEp-2 cell pattern classification. Cordelli and Soda [34] test four popular classifiers belonging to different paradigms: a multi-layer perceptron (MLP) as a neural network, a KNN as a statistical classifier, an SVM as a kernel machine, and AdaBoost as an ensemble of classifiers. In [31], the authors selected two ANN-based classifiers based on the MLPs and the radial basis

function (RBF) network architecture to separate intrinsically dubious samples whose error tolerance can be flexibly set. In [9], learning vector quantization (LVQ), which is a prototype-based supervised classification algorithm and can be understood as a special case of an ANN, utilized the normalized feature vector to differentiate the autoantibody fluorescence patterns.

Methods

The proposed classification architecture consists of four sequential steps: block segmentation, feature extraction, block pattern classification and well pattern classification. To our knowledge, block segmentation has never been used to process HEp-2 cell images. Various combinations of features and classifiers are utilized to identify the patterns of the blocks to explore the best features and classifiers suitable for this application. But not all combinations are used in the block pattern classification; for example, the VLFeat Package has its own classifier for the SIFT feature, so we just use this combination. As to the fusion rules, the weighted sum rule (WSR) is only defined in the KNN classifier, so in other classifiers, we just aggregate the block patterns to classify the staining pattern of the specimen image with WR and weighted majority rule (WMR) rules.

3.1 Block Segmentation

As mentioned above, the previous research into HEp-2 cell image segmentation mainly concentrated on cell segmentation, which also has drawbacks affecting the final sensitivity of the segmentation. For example, Otsu's thresholding method can choose the threshold to minimize the intra-class variance of the black and white pixels automatically, but due to the variety of ANA patterns, Otsu's algorithm always failed to segment cells of discrete speckled and nucleolar patterns and resulted in over-segmentation [8]. Even though two-stage watershed segmentation [10] uses two watershed transformations to avoid over-segmentation, it may occur in generating erroneous outlines of IIF cells because of noise and speckles in IIF images. Since the two-stage watershed segmentation uses a great number of morphological techniques, including pre-processing, Otsu's thresholding, region merging and region elimination, in the expectation of better segmentation performance, its time complexity and space complexity significantly enlarge in contrast to Otsu's segmentation method. The methods [8, 10, 35] proposed in the previous works for the segmentation utilized various techniques to eliminate over-segmentation and overlap problems, which have no effects on the performance of block segmentation although there are overlap areas between different blocks.

Block segmentation is much easier to implement than cell segmentation and does not have the same problems as cell segmentation. As is shown in Figure 3, first the RGB image is converted into a binary image and morphological erosion with a disk mask is performed; then the connected regions, which determine the position of candidate blocks, are located. The centre of the connected region is

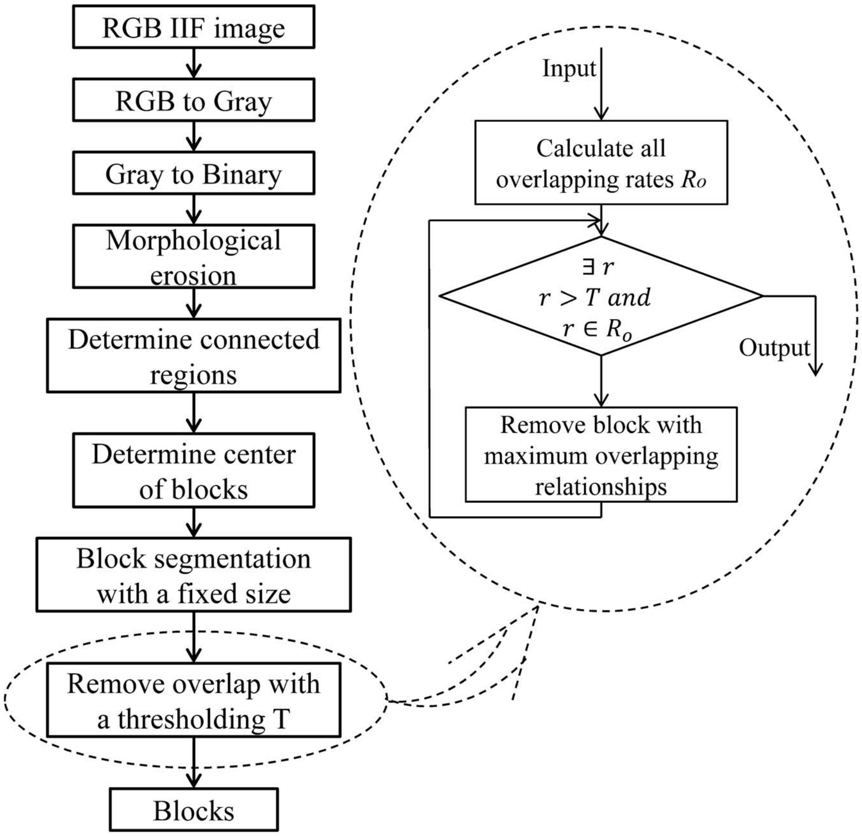

Figure 3. Flowchart of block segmentation.

doi:10.1371/journal.pone.0113132.g003

regarded as the centre of the block with a fixed size, such as and (the set depending on the size of the well image). The centre of the connected region is defined as (Figure 4a)

$$(x_m, y_m) = \left(\frac{x_{\max} - x_{\min}}{2}, \frac{y_{\max} - y_{\min}}{2}\right) \quad (1)$$

where (x_m, y_m) denotes the location of the centre and $x_{\max}$ and $x_{\min}$ denote the maximum and minimum x axes of the connected region. Similarly, $y_{\max}$ and $y_{\min}$ are the maximum and minimum y axes of the connected region.

Sometimes, the overlapping area occupies a large part of the total area, here using R_{ol} to present the rate of overlap areas between two blocks

$$R_{ol} = \frac{S_o}{S_1 + S_2 - S_o} \quad (2)$$

where S_o denotes the area of the overlapping area, S_1 denotes the area of one block and S_2 denotes the area of another block overlapping with the former block as shown in Figure 4b. Depending on the overlapping rates, the block with the maximum overlapping relationships is removed first and then the second maximum, then the third, and so on, until there is no overlapping rate which is

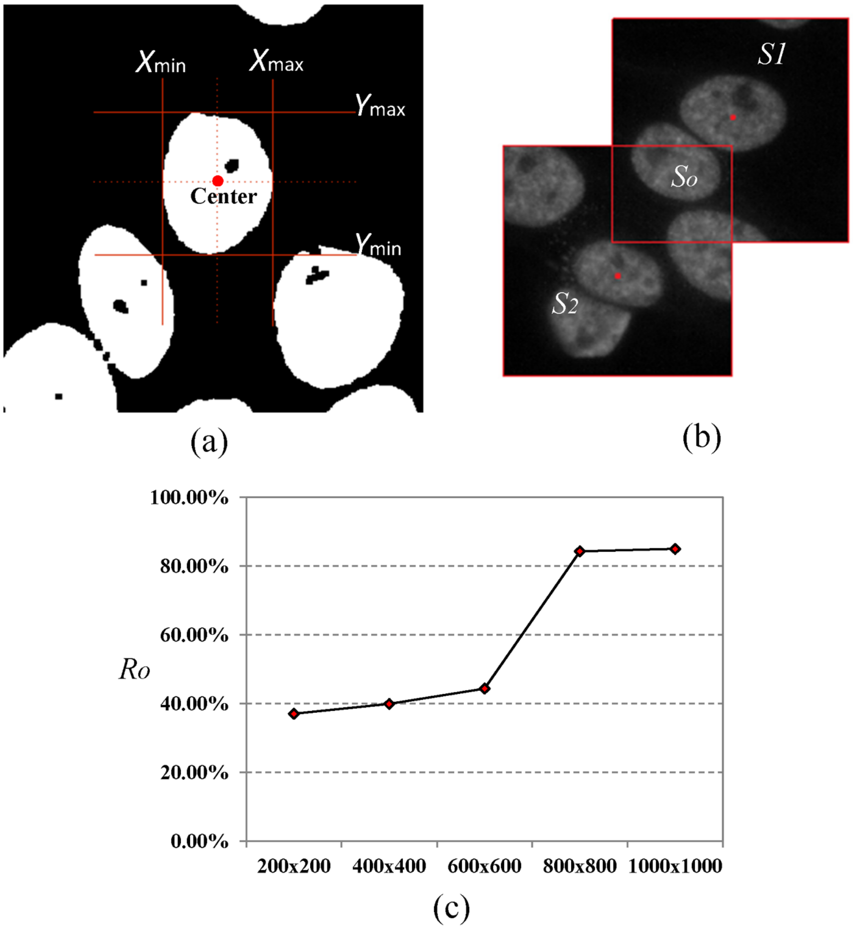

Figure 4. Overlap in the block segmentation: (a) calculate the centre of the connected regions; (b) overlap problem; (c) the overlapping rates with the size increasing.

doi:10.1371/journal.pone.0113132.g004

larger than the threshold. Here the overlapping relationship means the mapping from one block to another if they have overlaps. A block may overlap with many other blocks, so the block with the most overlapping relationships is removed first.

The preliminary experiments show that the average overlapping rate of two blocks increases as the block enlarges (Figure 4c). In order to decrease the number of blocks separated from an image and the influence of block overlap, one of the two overlapping blocks will be removed if the overlap rate is larger than 0.5. But if the block is too small, for example, 200×200 and 400×400 (Figure 4b), then most of the blocks are retained resulting in enormous experimental complexity and the blocks are too large to obtain sufficient blocks with a low overlapping rate, e.g. the average overlapping rate exceeds 80% when the size is 800×800 or 1000×1000. Moreover, the investigation and experiments demonstrate that the block of 600×600 is the most suitable for block segmentation and classification. The overlap problem in block segmentation has no effect on the final well pattern

classification as the blocks with overlapping relationships are all either in the training set or in the test set.

3.2 Feature Extraction

In this section, in total four practical features, LBP, SIFT, LDA and GLCM, are solely explored to identify fluorescence patterns. These features are briefly described as follows.

LBP Features

The original LBP operator, introduced by Ojala et al. [36], is a powerful means of texture description. Here, we use the notation LBP_P^R for the LBP operator (Figure 5a, 5b, 5c). The subscript represents using the operator in a (P, R) neighbourhood. A histogram H_i of the labelled image $I_l(x,y)$ can be defined as:

$$H_i = \sum_{x,y} L\{I_l(x,y) = i\} \quad i = 0,1,\ldots,n-1 \tag{3}$$

where n is the number of different labels produced by the LBP operator and

$$L\{A\} = \begin{cases} 1 & A \text{ is } \textit{true} \\ 0 & A \text{ is } \textit{false} \end{cases} \tag{4}$$

This histogram contains information about the distribution of the local micro-patterns, such as edges, spots and flat areas, over the whole image. For efficient

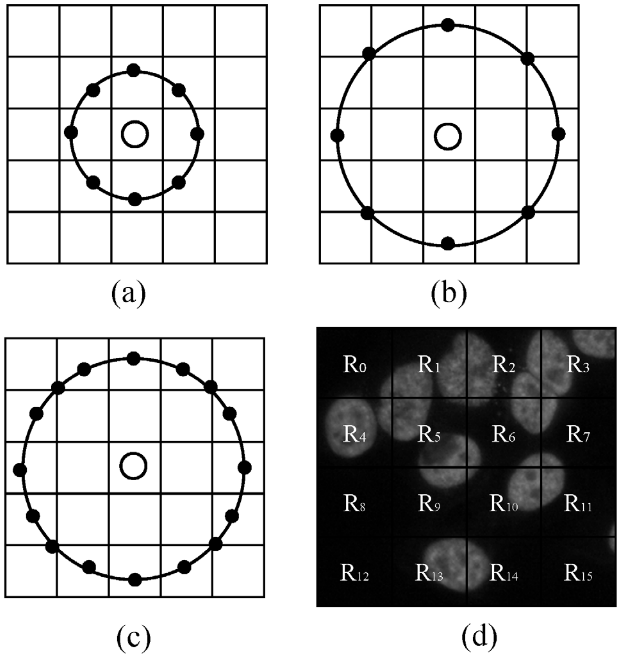

Figure 5. LBP descriptors: (a) LBP_8^1; (b) LBP_8^2; (c) LBP_{16}^2; (d) division for retaining spatial information.

doi:10.1371/journal.pone.0113132.g005

image feature representation, spatial information should be retained. So the image is divided into regions $R_0, R_1, \cdots\cdots, R_{m-1}$ (see Figure 5d, here m=16) and the spatially enhanced histogram is defined as [18]

$$H_{i,j} = \sum_{x,y} L\{I_l(x,y) = i\} * L\{(x,y) \in R_j\} \quad i = 0,1,\ldots,n-1; j = 0,1,\ldots,m-1 \quad (5)$$

In this experiment, the best LBP descriptor is LBP_8^2, and the dimension of the feature vector is 256.

SIFT Features

Lowe [13] summed up the existing feature detection method based on invariants technology in 2004, and formally proposed the SIFT algorithm invariant to common image transformations (image scaling, rotation, even affine transformation), illumination changes and noise. The SIFT algorithm first undertakes feature detection in scale space and defines the key point positions and the scale of the key points, and then uses the main direction of the neighbourhood gradient of the key points as the direction features of the points in order to achieve the operator independent of scale and direction. The MATALB Code S1 of SIFT we used is from http://www.vlfeat.org/index.html [37]. Different images have feature vectors with different dimensions, but each element has a direction parameter with 128 dimensions.

LDA Features

Principal component analysis (PCA) and LDA [38–40] are two powerful tools used for dimensionality reduction and feature extraction in most pattern recognition applications. Due to the number of blocks being so numerous that pattern classification only based on PCA characteristics may waste too much time, LDA features with five dimensions based on PCA characteristics are utilized to identify HEp-2 cells, achieving better performance than that only using PCA.

GLCM Features

GLCM [22] is a powerful technique that extracts texture characteristics from the spatial relationship between intensity values at specified offsets and reports the distribution of co-occurring values between local pixels based on different distances and angles. Here we extract 44 features, represented by the mean and the range value over the four GLCMs for each of the 22 statistical measures (e.g. correlation, cluster prominence, cluster shade, energy, entropy, variance, homogeneity and maximum probability, etc. [22]) in [16].

3.3 Block Pattern Classification

In this procedure, three commonly used classifiers, i.e. KNN using Euclidean distance, common BPNN with sigmoid units and SVM with linear kernel function, are used with different features for block recognition. Several different patterns may appear in a single image, but the segmentation method proposed

here only considers images with a unique pattern, which implies that blocks separated from an image are all marked with the pattern of the same well image.

The KNN classification algorithm, presented by Cover and Hart [28] in 1967, is a more mature approach in theory, but also one of the simplest machine learning algorithms. This decision rule provides a simple non-parametric procedure for the assignment of a class label to the input pattern based on the class labels represented by the k-closest neighbours of the vectors. BPNN learning methods provide a robust approach to approximating real-valued, discrete-valued, and vector-valued target functions. For certain types of problems, such as learning to interpret complex real-world sensor data, ANNs are among the most effective learning methods currently known. SVM is a powerful machine learning method successfully used in many applications and the classification is based on the implicit mapping of data to a higher dimensional space via a kernel function and on the identification of the maximum-margin hyperplane that separates the given training instances in this high-dimensional space [16].

3.4 Well Pattern Classification

To classify the screening patterns of the whole image into one of the basic classes mentioned in Section 1, first blocks should be segmented from the well image and then the set of features extracted; second the staining patterns of blocks labelled by the pattern of the original image are classified, and finally the staining pattern of the whole well is distinguished based on the results of the classification of its cells (Figure 6).

In fact, such an approach based on classification of individual blocks cannot detect the occurrence of multiple patterns since there may be cells with different patterns in a block marked with one pattern. But it is acceptable that most cells in a block belong to the class of the block. Furthermore, this approach is greatly tolerant and robust to misclassifications in block recognition since the final label of the whole image is aggregated by the classification information of all the blocks segmented from the image. Indeed, if enough blocks per well are available, it is reasonable that block misclassification, if limited, does not affect the final well pattern classification.

Typical fusion techniques, including majority rule (MR), WMR [41] and WSR [42, 43] (see Figure 7), will be used in this section to combine the results of block recognition. However, a critical point of these fusion rules is that different blocks belonging to the same well should be included in either the training set or the testing set, which guarantees that the final well pattern is determined by all the blocks belonging to this well image. So we randomly subdivided all the well images into two equal partitions and different blocks belonging to the same well were all in one partition. In the following, we briefly describe these fusion rules.

First, a conceptual formula [43] is given as follows:

$$WC_i = \sum_{B} \theta(b) \bullet I_i(b) \tag{6}$$

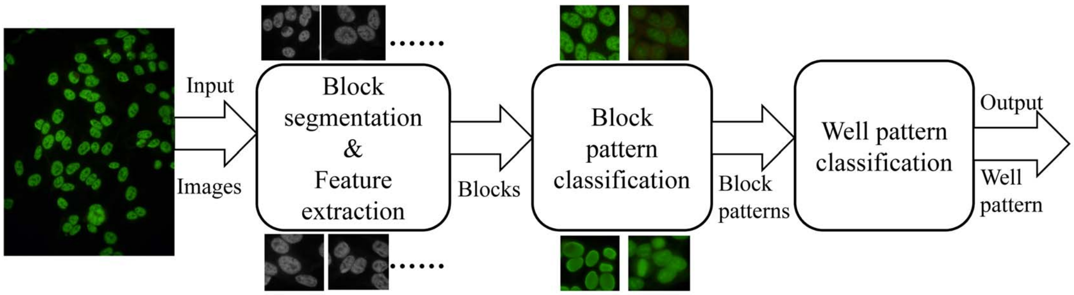

Figure 6. Architecture of well pattern classification.

doi:10.1371/journal.pone.0113132.g006

in which $\theta(b)$ indicates the weighted parameter of the input block b in the block set B and $I_i(b)$ is defined as follows:

$$I_i(b)=\begin{cases}1 & block\ b\in class\ C_i\\ 0 & otherwise\end{cases} \tag{7}$$

The index of the final class of the well staining pattern is the class for which WC_i is maximum. If $\theta(b)=1$, the rule is MR; if $\theta(b)$ indicates the number of cells in block b, the fusion rule is defined as WMR and if $\theta(b)$ indicates the classification reliability of the input block b, this rule becomes WSR, which is only used in the KNN classification.

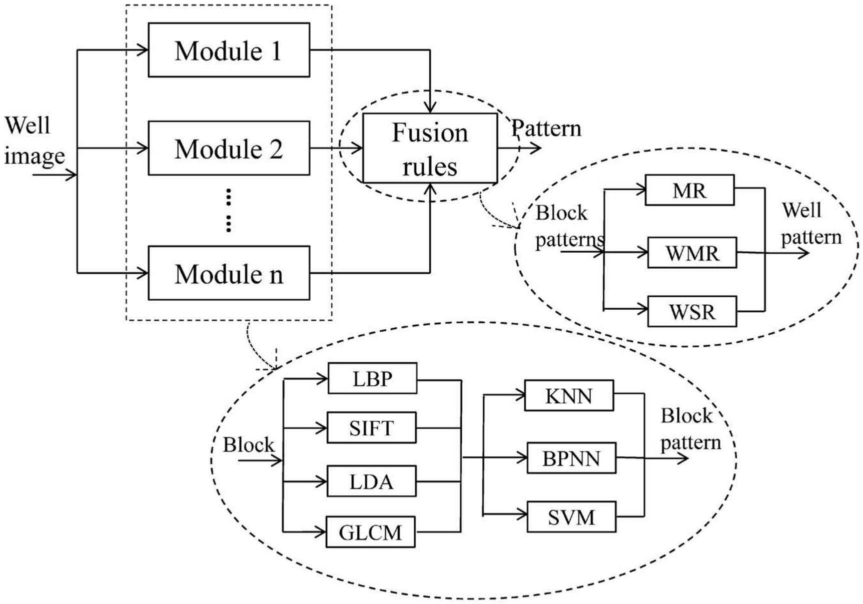

Figure 7. Method flow in the classification architecture.

doi:10.1371/journal.pone.0113132.g007

The classification reliability for the KNN [42] classifier is given by

$$\theta = min\left\{ max\left\{ 1 - \frac{O_{win}}{O_{max}} \right\}, 1 - \frac{O_{win}}{O_{win2}} \right\} \quad (8)$$

where O_{win} denotes the smallest distance of b from a reference sample belonging to the same pattern of b; O_{max} implies the highest among the values of O_{win} obtained for samples in a set disjointed from the reference set and the test set and O_{win2} is the distance between x and the reference sample with the second smallest distance from x among all the reference set samples belonging to a class which is different from that of O_{win}.

Experimental Results

Note that the proposed method to identify the staining pattern of the HEp-2 cell image here only considers images with a unique staining pattern; implying that blocks separated from an image are all marked with the pattern of the same well image. Not only the direct classification of the whole image, but also the staining pattern classification of the well image, based on cell segmentation as the control group, abides by this principle. Therefore, block segmentation is equal to cell segmentation in the problem to be solved.

4.1 Dataset

In this study, the IIF images were collected based on HEp-2 substrate at a serum dilution of 1:80. A physician takes images of slides with an acquisition unit consisting of the fluorescence microscope coupled with a commonly used fluorescence microscope (Axioskop 2, CarlZeiss, Jena, Germany) at 640-fold magnification. The IIF images were taken by an operator with a colour digital camera (E-330, Olympus, Tokyo, Japan). The digitized images were of 8-bit photometric resolution for each RGB colour channel with a resolution of 3136×2352 pixels [9]. This image database contains 260 samples belonging to four different patterns, i.e. coarse speckled (CS), fine speckled (FS), nucleolar (NU) and peripheral (PE). The number of samples in each pattern were 167 (CS), 20 (FS), 38 (NU) and 35 (PE), and the odd-numbered half of them were selected to belong to the training set, and the remainder were the test set (Table 1). If ANA testing detects any of the four patterns, the patients may have specific systemic autoimmune diseases. For example, if the test detected the CS pattern, the patients may have systemic lupus erythematosus (SLE), mixed connective tissue disease (MCTD), progressive systemic sclerosis (PSS) or cryoglobulinemia. Experiments have shown that the best features for ANA classification are LBP_8^2 features, which are shown in Figure 8.

The dataset is from the third party, Taichung Veterans General Hospital. The data is available upon request to the corresponding author. Moreover, the Code S1 of this experiment is uploaded in the online version for the convenience of

Table 1. The samples in the training set and in the testing set.

IIF patterns	Samples in the training set	Samples in the testing set
Coarse speckled	84	83
Fine speckled	10	10
Nucleolar	19	19
Peripheral	17	18
Total	130	130

doi:10.1371/journal.pone.0113132.t001

testing our proposed method on other datasets, please refer to Supporting Information: Instruction S1.

4.2 Direct Whole Image Pattern Classification

LBP descriptors of the HEp-2 images without cell segmentation and block segmentation were directly extracted, and then classified on the test set. It was found that the KNN classifier with the LBP_8^2 descriptor just achieved the best performance with a total accuracy of 83.08%, as depicted in Table 2; in particular, only half of the samples with peripheral patterns were classified into the right class, demonstrating that global classification is not applicable in some patterns.

4.3 Classification Based on Cell Segmentation

HEp-2 cell images were separated by Otsu's thresholding method and all cells divided from an image belonging to the training set or the test set were still regarded as the training set or the test set respectively. Subsequently, various combinations of classifiers and features were applied to the HEp-2 cells dataset, and then suitable fusion rules were used to aggregate the results of cell pattern classification into well pattern classification. The experiments showed that when utilizing a combination of KNN, LBP and MR, the total accuracy of the four distinct patterns is 90.77% (see Table 3), which is better than that in the direct classification. Compared with direct classification, the accuracy of the CS pattern increased to 100%, while that of the FS and NU patterns slightly decreased.

4.4 Classification Based on Block Segmentation

To better illustrate the advantages of block segmentation, such comparisons are described as follows: (a) the block segmentation considered is significantly different from cell segmentation, that is, the complexity of block segmentation is significantly lower than that of cell segmentation with numerous morphological techniques, and the block segmentation method just depends on connected regions; (b) in contrast to direct whole image classification, the classification based on block segmentation has a robust tolerance to misclassifications in the block recognition since the final label of the whole image is aggregated by classification information of all blocks segmented from the image. However, that

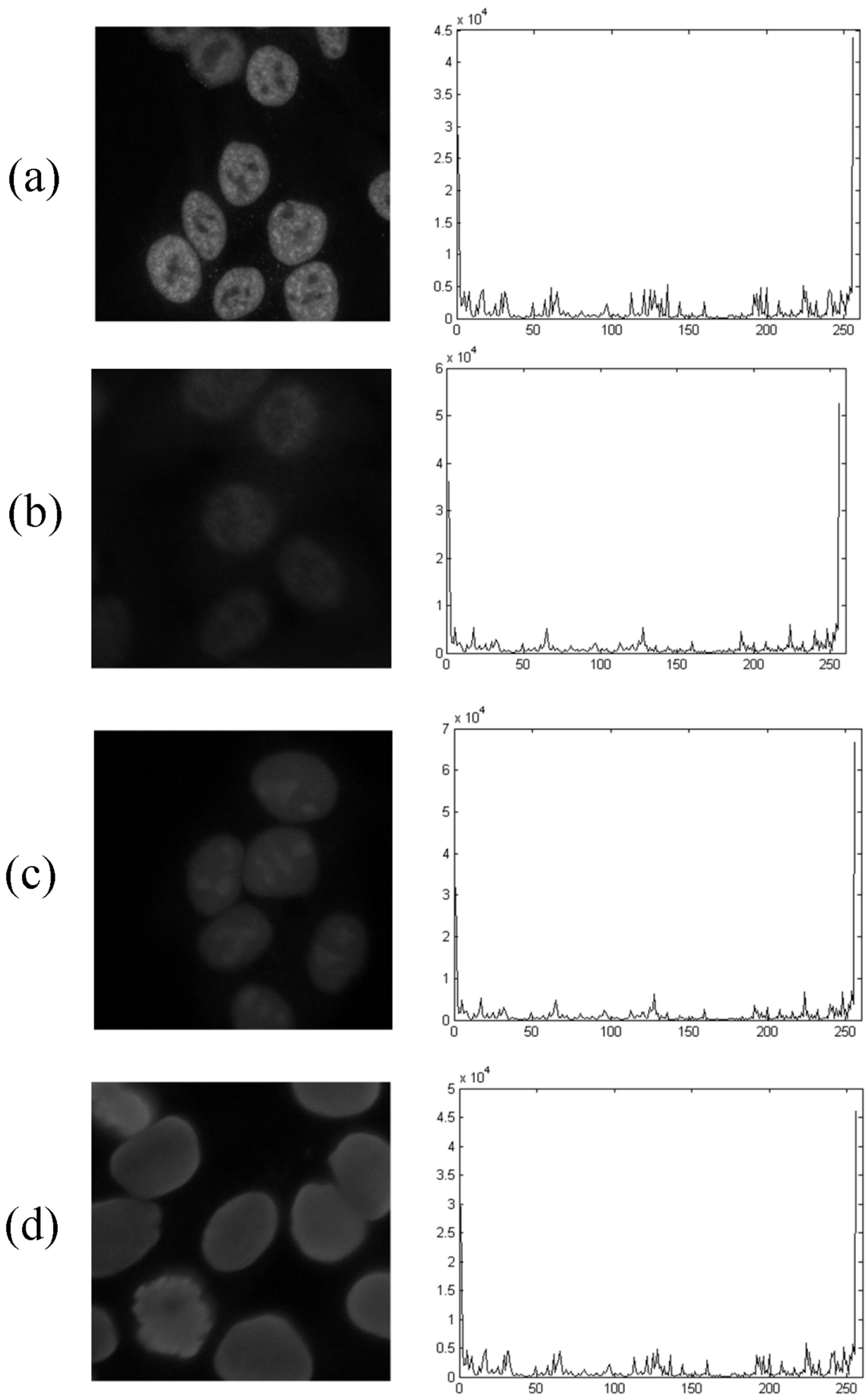
(a)
(b)
(c)
(d)
x 10^4
0
50
100
150
200
250

Figure 8. Four staining patterns and the corresponding LBP_8^2 descriptors: (a) coarse speckled (CS); (b) fine speckled (FS); (c) nucleolar (NU); (d) peripheral (PE).

doi:10.1371/journal.pone.0113132.g008

the size of the block requires a great number of explorations to determine, since there has been no regularity so far, is a problem.

In this experiment, various combinations of classifier, feature and fusion rule were utilized to evaluate the performance of the staining pattern recognition of the HEp-2 cell image. Figure 9 presents the accuracies of 10 combinations mainly focusing on the LBP feature and KNN classifier, with some passive combinations omitted, such as LDA feature and BPNN classifier, LDA feature and SVM classifier etc. LBP+BPNN+MR and LBP+KNN+WSR achieve the same accuracy, 94.62%, and the classification results based on the LBP feature is more positive than other features with a maximum accuracy of 76.15% using GLCM+KNN+MR (Figure 9). This indicates that the LBP feature is the most suitable characteristic to identify ANA patterns.

Two methods have the same accuracy, LBP+BPNN+MR and LBP+KNN+WSR, but their individual results for cell pattern classification are different. The latter, with a total accuracy of 82.21%, is slightly better than the former with 79.95%, as shown in Table 4. We used different fusion rules to aggregate the different classification results of the block pattern, but achieved the same positive performance, demonstrating the robust tolerance to misclassifications of well pattern classification based on the results of block pattern classification.

Even though only approximately half of the blocks marked with the peripheral pattern are correctly distinguished (Table 4) in the block pattern classification with the LBP characteristic and KNN classifier, there are still 12 samples among the test set of well images (18 samples) correctly classified (Table 5). Even though the accuracy of block pattern classification with the LBP feature and BPNN classifier is no more than 80%, the final well pattern classification based on it achieved positive performance with a total accuracy of 94.62% (Figure 9). In contrast with the classification based on cell segmentation (Table 3), the accuracies of FS and NU patterns both in well classification based on block segmentation reached 100% while those of CS and PE patterns slightly decreased.

Table 2. Classification results based on the whole image.

	CS	FS	NU	PE	Accuracy
CS	75	0	1	7	90.36%
FS	0	9	0	1	90.00%
NU	3	0	15	1	78.95%
PE	8	0	1	9	50.00%
Total					83.08%

doi:10.1371/journal.pone.0113132.t002

Table 3. Classification results based on cell segmentation with KNN classifier, LBP feature and Majority Rule.

	CS	FS	NU	PE	Accuracy
CS	83	0	0	0	100.0%
FS	2	5	3	0	70.00%
NU	3	0	14	2	73.68%
PE	2	0	0	16	77.78%
Total					90.77%

doi:10.1371/journal.pone.0113132.t003

Consequently, the performance of well classification based on block segmentation is a little better than that based on cell segmentation.

Moreover, mean class accuracy (MCA) is commonly used as the evaluation criteria in cell level classification. So here we use it to measure the performance of cell segmentation and block segmentation under the same circumstance, that is, feature, classifier and fusion rule. MCA can be defined as follows:

$$MCA = \frac{1}{K}\sum_{k=1}^{K} CCR_k \tag{9}$$

where CCR_k is the correct classification rate for class k and K is equal to the number of classes.

Table 6 reports the classification results of the proposed approach and some previous methods. The result of the classification based on block segmentation with MCA of 91.37% is significantly better than that of the others. Furthermore, some previous approaches, such as HOG and SVM, GLCM and SVM, are distinctly inapplicable in this dataset, achieving passive and biased accuracies.

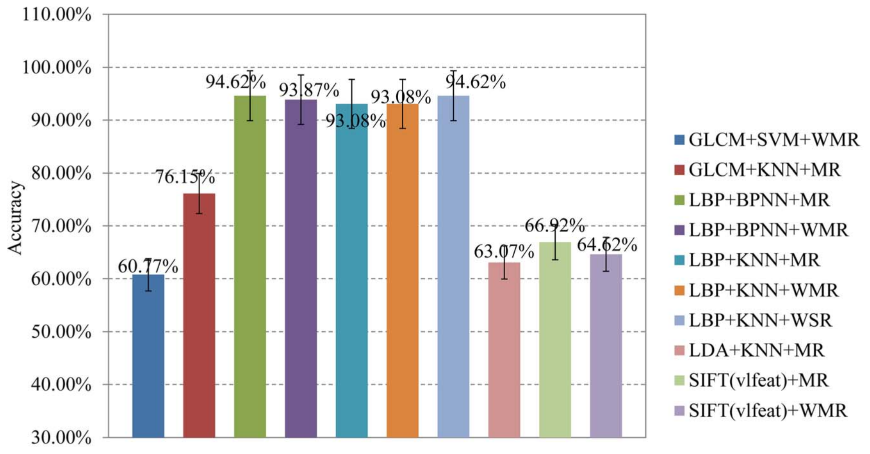

Figure 9. Accuracies of different combinations of classifier, feature and fusion rule: from right to left sequentially GLCM+SVM+WMR, GLCM+KNN+MR, LBP+BPNN+MR, LBP+BPNN+WMR, LBP+KNN+MR, LBP+KNN+WMR, LBP+KNN+WSR, LDA+KNN+MR, SIFT(vlfeat)+MR and SIFT(vlfeat)+WMR.

doi:10.1371/journal.pone.0113132.g009

Table 4. Classification results of block pattern classification of LBP+KNN and LBP+BPNN.

Block patterns	Ntrain	Ntest	Nc (LBP+BPNN)	Nc (LBP+KNN)
CS	1714	1584	1417(89.46%)	1458(92.05%)
FS	130	133	106(79.70%)	127(95.49%)
NU	419	440	239(54.32%)	321(72.95%)
PE	373	401	283(70.57%)	197(49.13%)
Total	2636	2558	79.95%	82.21%

Ntrain: number of blocks in the training set.
Ntest: number of blocks in the testing set.
Nc: number of correct classification of blocks.

doi:10.1371/journal.pone.0113132.t004

Conclusion and Discussion

In this study, in contrast to cell segmentation a new block segmentation method never used was proposed to process the original HEp-2 images and then classification of the block patterns was undertaken based on various selected features (GLCM, LBP, SIFT and LDA) and classifiers (KNN, BPNN and SVM), commonly used in the previous studies of cell pattern classification. Subsequently, fusion rules (MR, WMR, and WSR) were used to aggregate the results of the block pattern classification to identify the staining patterns of the whole images. The results show that the proposed method can classify the well images correctly with an accuracy of 94.62% depending on the combination of LBP, KNN and MSR or the combination of LBP, BPNN and MR, which is better than pattern classification with a total accuracy of 90.77% based on cell segmentation and direct whole image classification with a total accuracy of 83.08%.

The block segmentation considered is significantly different from cell segmentation, that is, the complexity of block segmentation is significantly lower than that of cell segmentation with numerous morphological techniques, and the block segmentation method just depends on connected regions. In contrast to direct whole image classification, the classification based on block segmentation has a robust tolerance to misclassifications in block recognition since the final label of the whole image is aggregated by classification information of all the blocks segmented from the image. However, that the size of the block requires a great number of explorations to determine, since there has been no regularity so

Table 5. Classification results based on block segmentation with KNN classifier, LBP feature and Weighted Sum Rule.

	CS	FS	NU	PE	Accuracy
**CS**	82	0	1	0	98.80%
**FS**	0	10	0	0	100.0%
**NU**	0	0	19	0	100.0%
**PE**	5	0	1	12	66.67%
**Total**					94.62%

doi:10.1371/journal.pone.0113132.t005

Table 6. Comparison of direct whole image classification, classification based on cell segmentation and classification based on block segmentation.

	Total accuracy	Mean class accuracy
Direct whole image classification	83.08%	77.33%
Cordelli E [34] (LBP and KNN) and WSR	90.77%	78.14%
Cordelli E [34] (LBP and KNN) and MR	90.00%	79.03%
Cordelli E [34] (LBP and SVM) and MR	63.85%	25.00%
Ghosh S [24] (Texture feature, HOG and SVM) and MR	58.46%	22.89%
Di Cataldo S [16] (GLCM and SVM) and MR	59.23%	23.19%
Classification based on block segmentation	94.62%	91.37%

doi:10.1371/journal.pone.0113132.t006

far, is a problem. If the block size is too large, block segmentation will waste too much memory compared with cell segmentation. Moreover, well pattern classification based on the classification of individual blocks cannot detect the occurrence of multiple patterns since there may be cells with different patterns in a block marked with one pattern. However, this approach is greatly tolerant and robust to misclassifications in block recognition. If enough blocks per well are available, it is reasonable that block misclassification does not affect the final well pattern classification.

Supporting Information

Instruction S1

doi:10.1371/journal.pone.0113132.s001 (PDF)

Code S1

doi:10.1371/journal.pone.0113132.s002 (ZIP)

Acknowledgments

I would particularly like to thank Kuo-Kun Tseng and Huang-Nan Huang, who have suggested numerous improvements to both the content and presentation of this paper. In addition, I would like to thank Taichung Veterans General Hospital for their suggestions and supporting the ANA database.

Author Contributions

Conceived and designed the experiments: KT ZH CY. Performed the experiments: JL. Analyzed the data: JL KT HH. Contributed reagents/materials/analysis tools: ZH CY. Wrote the paper: JL KT HH. Edited the paper: ZH CY.

References

1. **Davidson A, Diamond B** (2001) Autoimmune diseases. N Engl J Med 345: 340–350.

2. **Fritzler MJ** (2008) Challenges to the use of autoantibodies as predictors of disease onset, diagnosis and outcomes. Autoimmunity Reviews 7: 616–620.
3. **Worman HJ, Courvalin JC** (2003) Antinuclear antibodies specific for primary biliary cirrhosis. Autoimmunity Reviews 2: 211–217.
4. **Huang YC, Hsieh TY, Chang CY, Cheng WT, Lin YC** (2012) HEp-2 cell images classification based on textural and statistic features using self-organizing map. In: Pan J-S, Chen S-M, Nguyen N, editors. Intelligent Information and Database Systems: Springer Berlin Heidelberg. pp. 529–538.
5. **Iannello G, Onofri L, Soda P** (2012) A bag of visual words approach for centromere and cytoplasmic staining pattern classification on HEp-2 images. Computer-Based Medical Systems (CBMS), 2012 25th International Symposium on. pp. 1–6.
6. **Rigon A, Soda P, Zennaro D, Iannello G, Afeltra A** (2007) Indirect immunofluorescence in autoimmune diseases: Assessment of digital images for diagnostic purpose. Cytometry Part B: Clinical Cytometry 72: 472–477.
7. **Creemers C, Guerti K, Geerts S, Van Cotthem K, Ledda A, et al.** (2011) HEp-2 cell pattern segmentation for the support of autoimmune disease diagnosis. Proceedings of the 4th International Symposium on Applied Sciences in Biomedical and Communication Technologies. pp. 28.
8. **Huang YL, Jao YL, Hsieh TY, Chung CW** (2008) Adaptive automatic segmentation of HEp-2 cells in indirect immunofluorescence images. Sensor Networks, Ubiquitous and Trustworthy Computing, 2008. SUTC'08. IEEE International Conference on. pp. 418–422.
9. **Hsieh TY, Huang YC, Chung CW, Huang YL** (2009) HEp-2 cell classification in indirect immunofluorescence images. Information, Communications and Signal Processing, 2009. ICICS 2009. 7th International Conference on. pp. 1–4.
10. **Huang YL, Chung CW, Hsieh TY, Jao YL** (2008) Outline detection for the HEp-2 cell in indirect immunofluorescence images using watershed segmentation. Sensor Networks, Ubiquitous and Trustworthy Computing, 2008. SUTC'08. IEEE International Conference on. pp. 423–427.
11. **Soares F, Muge F** (2004) Watershed lines suppression by waterfall marker improvement and line-neighbourhood analysis. Pattern Recognition, 2004. ICPR 2004. Proceedings of the 17th International Conference on. pp. 604–607.
12. **Snell V, Christmas W, Kittler J** (2012) Texture and shape in fluorescence pattern identification for auto-immune disease diagnosis. Pattern Recognition (ICPR), 2012 21st International Conference on. pp. 3750–3753.
13. **Lowe DG** (2004) Distinctive image features from scale-invariant keypoints. International Journal of Computer Vision 60: 91–110.
14. **Wang H, Yang K, Gao F, Li J** (2011) Normalization methods of SIFT vector for object recognition. Distributed Computing and Applications to Business, Engineering and Science (DCABES), 2011 Tenth International Symposium on. pp. 175–178.
15. **Sorwar G, Abraham A, Dooley LS** (2001) Texture classification based on DCT and soft computing. Fuzzy Systems, 2001. The 10th IEEE International Conference on. pp. 545–548.
16. **Di Cataldo S, Bottino A, Ficarra E, Macii E** (2012) Applying textural features to the classification of HEp-2 cell patterns in IIF images. Pattern Recognition (ICPR), 2012 21st International Conference on. pp. 3349–3352.
17. **Kuan L, Jianping Y, Zhi L, Xiangfei K, Rui Z, et al.** (2012) Multiclass boosting SVM using different texture features in HEp-2 cell staining pattern classification. Pattern Recognition (ICPR), 2012 21st International Conference on. pp. 170–173.
18. **Ahonen T, Hadid A, Pietikainen M** (2006) Face description with local binary patterns: Application to face recognition. Pattern Analysis and Machine Intelligence, IEEE Transactions on 28: 2037–2041.
19. **Guo Z, Zhang L, Zhang D** (2010) A completed modeling of local binary pattern operator for texture classification. Image Processing, IEEE Transactions on 19: 1657–1663.
20. **Ojala T, Pietikainen M, Maenpaa T** (2002) Multiresolution gray-scale and rotation invariant texture classification with local binary patterns. Pattern Analysis and Machine Intelligence, IEEE Transactions on 24: 971–987.
21. **Ersoy I, Bunyak F, Peng J, Palaniappan K** (2012) HEp-2 cell classification in IIF images using shareboost. pattern recognition (ICPR), 2012 21st International Conference on. pp. 3362–3365.

22. **Haralick RM, Shanmugam K, Dinstein IH** (1973) Textural features for image classification. Systems, Man and Cybernetics, IEEE Transactions on: 610–621.

23. **Strandmark P, Ulén J, Kahl F** (2012) Hep-2 staining pattern classification. Pattern Recognition (ICPR), 2012 21st International Conference on. pp. 33–36.

24. **Ghosh S, Chaudhary V** (2012) Feature analysis for automatic classification of HEp-2 florescence patterns: Computer-aided diagnosis of auto-immune diseases. Pattern Recognition (ICPR), 2012 21st International Conference on. pp. 174–77.

25. **Yang Y, Wiliem A, Alavi A, Hobson P** (2013) Classification of human epithelial type 2 cell images using independent component analysis. ICIP, pp. 733–737.

26. **Wiliem A, Wong Y, Sanderson C, Hobson P, Chen S, et al.** (2013) Classification of human epithelial type 2 cell indirect immunofluoresence images via codebook based descriptors. arXiv preprint arXiv: 13041262.

27. **Thibault G, Angulo J** (2012) Efficient statistical/morphological cell texture characterization and classification. Pattern Recognition (ICPR), 2012 21st International Conference on. pp. 2440–2443.

28. **Cover T, Hart P** (1967) Nearest neighbor pattern classification. Information Theory, IEEE Transactions on 13: 21–27.

29. **Fukunaga K, Narendra PM** (1975) A branch and bound algorithm for computing k-nearest neighbors. Computers, IEEE Transactions on 100: 750–753.

30. **Soda P, Iannello G** (2006) A multi-expert system to classify fluorescent intensity in antinuclear autoantibodies testing. Computer-Based Medical Systems, 2006. CBMS 2006. 19th IEEE International Symposium on. pp. 219–224.

31. **Soda P, Iannello G** (2006) ANN-based classification of indirect immunofluorescence images. World Academy of Science, Engineering and Technology. 2: 829–834.

32. **Soda P, Iannello G** (2007) A hybrid multi-expert system for HEp-2 staining pattern classification. Image Analysis and Processing, 2007. ICIAP 2007. 14th International Conference on. pp. 685–690.

33. **Soda P, Iannello G, Vento M** (2009) A multiple expert system for classifying fluorescent intensity in antinuclear autoantibodies analysis. Pattern Analysis and Applications 12: 215–226.

34. **Cordelli E, Soda P** (2011) Color to grayscale staining pattern representation in IIF. Computer-Based Medical Systems (CBMS), 2011 24th International Symposium on. pp. 1–6.

35. **Cheng CC, Hsieh TY, Taur JS, Chen YF** (2013) An automatic segmentation and classification framework for anti-nuclear antibody images. BioMedical Engineering OnLine 12: S5.

36. **Ojala T, Pietikäinen M, Harwood D** (1996) A comparative study of texture measures with classification based on featured distributions. Pattern recognition 29: 51–59.

37. **Vedaldi A, Fulkerson B** (2010) VLFeat: An open and portable library of computer vision algorithms. Proceedings of the international conference on Multimedia. pp. 1469–1472.

38. **Sahoolizadeh AH, Heidari BZ, Dehghani CH** (2008) A new face recognition method using PCA, LDA and neural network. International Journal of Computer Science and Engineering 2: 218–223.

39. **Wold S, Esbensen K, Geladi P** (1987) Principal component analysis. Chemometrics and Intelligent Laboratory Systems 2: 37–52.

40. **Zhang D, Jing XY, Yang J** (2006) Linear discriminant analysis. D Zhang, X Jing, Jing, & J Yang, (Eds), Biometric Image Discrimination Technologies: Computational Intelligence and Its Applications Series: 41–64.

41. **Kittler J, Hatef M, Duin RP, Matas J** (1998) On combining classifiers. Pattern Analysis and Machine Intelligence, IEEE Transactions on 20: 226–239.

42. **Cordella LP, Foggia P, Sansone C, Tortorella F, Vento M** (1999) Reliability parameters to improve combination strategies in multi-expert systems. Pattern Analysis & Applications 2: 205–214.

43. **Soda P, Iannello G** (2009) Aggregation of classifiers for staining pattern recognition in antinuclear autoantibodies analysis. Information Technology in Biomedicine, IEEE Transactions on 13: 322–329.
